# Supplementary material for: Comparative Analysis of the Symbiotic Microbiota in the Chinese Mitten Crab (Eriocheir sinensis): Microbial Structure, Co-Occurrence Patterns, and Predictive Functions
Source: Microorganisms. 2023 Feb 21;11(3):544. doi: 10.3390/microorganisms11030544 (PMC10053967; doi:10.3390/microorganisms11030544)
Supplement: Supplementary file 1 [file microorganisms-11-00544-s001.zip › microorganisms-2173236-supplementary.docx]

**Supporting Information:**

**Comparative analysis of the symbiotic microbiota in Chinese mitten crab (*Eriocheir sinensis*): microbial structure, co-occurrence patterns, and predictive functions**

Jicheng Yang ^1,2^, Qianqian Zhang ^1,5^, Tanglin Zhang^1^, Shuyi Wang ^1,4^, Jingwen Hao^1,4^, Zhenbing Wu ^3*^, Aihua Li ^1,4,5*^

^1^ State Key Laboratory of Freshwater Ecology and Biotechnology, Institute of Hydrobiology, Chinese Academy of Sciences, Wuhan 430072, China

^2^ College of Fisheries and Life Science, Dalian Ocean University, Dalian 116023, China

^3^School of Environmental Science and Engineering, Huazhong University of Science and Technology, Wuhan 430074, China

^4^University of Chinese Academy of Sciences, Beijing 100049, China

^5^National Aquatic Biological Resource Center, NABRC, Wuhan 430072, China

* Corresponding authors:

Aihua Li, E-mail address: liaihua@ihb.ac.cn, Tel: 86-27-68780053, ORCID: 0000-0003-0867-9823

Zhenbing Wu, E-mail address: wuzhenbing@ihb.ac.cn, ORCID: 0000-0002-3424-5079

**Table S1.** Crab collection information.

| Sample number | Location | Gender | Size/g | Time |
| --- | --- | --- | --- | --- |
| 1 | Beihe | Male | 156.17 | Dec. 4th, 2021 |
| 2 | Beihe | Male | 144.44 | Dec. 4th, 2021 |
| 3 | Beihe | Male | 163.26 | Dec. 4th, 2021 |
| 4 | Beihe | Male | 156.25 | Dec. 4th, 2021 |
| 5 | Beihe | Female | 166.25 | Dec. 4th, 2021 |
| 6 | Beihe | Female | 151.21 | Dec. 4th, 2021 |
| 7 | Diaochahu | Male | 159.17 | Dec. 4th, 2021 |
| 8 | Diaochahu | Male | 175.17 | Dec. 4th, 2021 |
| 9 | Diaochahu | Male | 163.26 | Dec. 4th, 2021 |
| 10 | Diaochahu | Male | 151.14 | Dec. 4th, 2021 |
| 11 | Diaochahu | Male | 155.22 | Dec. 4th, 2021 |
| 12 | Diaochahu | Male | 170.08 | Dec. 4th, 2021 |

**Table S2.** Summary of alpha diversity indices and the number of taxa calculated based on a cutoff of 97% similarity of 16S rRNA sequences in different sites. B: hemolymph (B1–B12); H: hepatopancreas (H1–H12); I: intestine (I1–I12).

| Indices | Group | | |
| --- | --- | --- | --- |
|  | B | H | I |
| Sobs | 2593.75 ± 600.23 | 3049.08 ± 778.26 | 1780.33 ± 406.52 |
| Chao 1 | 3550.30 ± 502.41 | 3843.84 ± 599.24 | 2812.84 ± 463.57 |
| Shannon | 6.24 ± 0.28 | 5.84 ± 1.60 | 3.37 ± 0.56 |
| Simpson | 0.01 ± 0.00 | 0.08 ± 0.12 | 0.12 ± 0.06 |
| Number of phyla | 59 | 64 | 57 |
| Number of classes | 169 | 194 | 175 |
| Number of orders | 430 | 483 | 411 |
| Number of families | 744 | 853 | 696 |
| Number of genera | 1616 | 1919 | 1443 |
| Number of OTUs | 10575 | 14038 | 7664 |
| Coverage | 98.17 ± 0.18% | 98.39 ± 0.28% | 98.25 ± 0.27% |

**Table S3.** Analysis of similarity (ANOSIM) of the structure and function of the bacterial communities at different sites based on the Bray-Curtis metric. Permutation N = 999; R is assessed by permuting the grouping vector to obtain the empirical distribution of R under the null model; a *p*-value less than 0.05 means significant. B: hemolymph (B1–B12); H: hepatopancreas (H1–H12); I: intestine (I1–I12).

| Group | Community structure | | Community function | |
| --- | --- | --- | --- | --- |
|  | R | *p* | R | *p* |
| Whole comparison | 0.67 | 0.001 | 0.21 | 0.001 |
| B VS.H | 0.09 | 0.018 | 0.01 | 0.325 |
| H VS.I | 0.85 | 0.001 | 0.20 | 0.003 |
| B VS.I | 0.96 | 0.001 | 0.33 | 0.001 |

**Table S4.** Comparison of dominant phyla (> 1%, average relative abundance in all samples) in the bacterial communities of different sites. The same letter indicates no significant differences (*p* > 0.05) at different sites, while the different letter indicates significant differences (*p* < 0.05 or *p* <0.01) at different sites. B: hemolymph (B1–B12); H: hepatopancreas (H1–H12); I: intestine (I1–I12).

| Phyla | Group | | |
| --- | --- | --- | --- |
|  | B | H | I |
| Proteobacteria | 34.74% ± 5.26% a | 34.71% ± 18.42% a | 29.5% ± 18.42% a |
| Firmicutes | 15.10% ± 2.06% a | 25.73% ± 16.10% ab | 41.37% ± 23.49% b |
| Bacteroidota | 9.35% ± 2.87% a | 8.18% ± 3.84% ab | 23.06% ± 10.61% b |
| Acidobacteriota | 10.63% ± 2.24% a | 9.23% ± 3.37% a | 1.98% ± 0.96% b |
| Actinobacteriota | 6.06% ± 1.66% a | 4.72% ± 2.35% b | 0.94% ± 0.41% c |
| Chloroflexi | 3.75% ± 2.05% a | 3.23% ± 1.52% a | 0.49% ± 0.21% b |
| unclassified_norank_Bacteria | 4.4% ± 4.87% a | 1.54% ± 1.31% ab | 0.17% ± 0.11% b |
| Gemmatimonadota | 2.23% ± 0.68% a | 1.85% ± 0.81% a | 0.33% ± 0.15% b |
| Verrucomicrobiota | 1.96% ± 0.44% a | 1.34% ± 0.68% b | 0.19% ± 0.15% c |
| Patescibacteria | 1.09% ± 0.30% a | 1.25% ± 0.57% a | 0.52% ± 0.47% b |
| Desulfobacterota | 1.45% ± 0.73% a | 1.05% ± 0.63% a | 0.12% ± 0.10% b |
| Myxococcota | 1.25% ± 0.74% a | 0.82% ± 0.28% a | 0.17% ± 0.09% b |
| Planctomycetota | 0.97% ± 0.54% a | 1.06% ± 0.42% a | 0.11% ± 0.08% b |
| Fusobacteriota | 0.84% ± 0.44% a | 0.63% ± 0.38% a | 0.16% ± 0.09% b |
| Cyanobacteria | 0.83% ± 0.50% a | 0.70% ± 0.49% a | 0.09% ± 0.10% b |
| Nitrospirota | 0.79% ± 0.33% a | 0.54% ± 0.27% a | 0.15% ± 0.07% a |
| Methylomirabilota | 0.67% ± 0.29% a | 0.60% ± 0.23% a | 0.18% ± 0.09% b |
| Campilobacterota | 0.53% ± 0.63% a | 0.50% ± 0.42% a | 0.05% ± 0.04% a |
| SAR324_cladeMarine_group_B | 0.33% ± 0.38% a | 0.09% ± 0.11% b | 0.01% ± 0.01% b |
| others | 3.03% ± 0.55% a | 2.23% ± 0.91% b | 0.41% ± 0.19% c |

**Table S5.** Comparison of dominant genera (> 1%, average relative abundance in all samples) in the bacterial communities of different sites. The same letter indicates no significant differences (*p* > 0.05) at different sites, while the different letter indicates significant differences (*p* < 0.05 or *p* <0.01) at different sites. B: hemolymph (B1–B12); H: hepatopancreas (H1–H12); I: intestine (I1–I12).

| Genus | B | H | I |
| --- | --- | --- | --- |
| *Candidatus Bacilloplasma* | 0.35%±±0.71% a | 0.67%±0.68% a | 22.17%±19.69% b |
| *Candidatus Hepatoplasma* | 0.08%±0.07% a | 11.27%±18.22% b | 3.28%±6.11% ab |
| unclassified_Alphaproteobacteria | 0.12%±0.09% a | 0.18%±0.07% a | 9.53%±12.91% b |
| *Roseimarinus* | 0.06%±0.08% a | 0.09%±0.05% a | 8.89%±7.60% b |
| *Acinetobacter* | 0.82%±0.44% a | 0.67%±0.47% a | 4.68%±10.42% a |
| norank_Bacteria | 4.40%±4.87% a | 1.54%±1.31% b | 0.17%±0.11% b |
| *Vibrio* | 0.80%±1.95% a | 0.90%±1.62% a | 4.22%±5.45% b |
| *Shewanella* | 0.18%±0.49% a | 4.14%±12.54% a | 1.47%±2.30% a |
| *Dysgonomonas* | 0.06%±0.11% a | 0.06%±0.04% a | 5.46%±4.00% b |
| *Enterobacter* | 2.94%±2.55% a | 2.27%±1.95% ab | 0.26%±0.24% b |
| *Bacteroides* | 0.77%±0.32% a | 0.49%±0.28% a | 3.86%±4.78% b |
| *ZOR0006* | 0.10%±0.17% a | 0.13%±0.12% a | 4.70%±2.32% b |
| norank_Mycoplasmataceae | 0.02%±0.03% a | 0.22%±0.22% a | 3.55%±4.79% b |
| *Enterococcus* | 1.40%±1.32% a | 2.27%±3.40% a | 0.10%±0.14% a |
| norank_Vicinamibacterales | 1.87%±0.71% a | 1.47%±0.49% a | 0.42%±0.18% b |
| *Pseudomonas* | 1.92%±1.09% a | 1.47%±1.19% a | 0.31%±0.38% b |
| *Aeromonas* | 0.17%±0.27% a | 3.04%±9.48% a | 0.30%±0.36% a |
| *RB41* | 1.55%±0.74% a | 1.57%±0.86% a | 0.34%±0.19% b |
| *Ralstonia* | 1.74%±1.90% a | 1.55%±1.45% ab | 0.16%±0.21% b |
| *Streptococcus* | 1.39%±0.65% a | 1.49%±0.78% a | 0.51%±0.21% b |
| norank_Vicinamibacteraceae | 1.32%±0.41% a | 1.11%±0.34% a | 0.34%±0.12% b |
| *Flavobacterium* | 0.13%±0.15% a | 0.21%±0.20% a | 2.35%±5.24% a |
| *Tyzzerella* | 0.08%±0.12% a | 0.05%±0.05% a | 2.49%±2.58% b |
| *Pragia* | 0.07%±0.14% a | 0.03%±0.03% a | 2.47%±2.54% b |
| *Lactobacillus* | 1.55%±0.81% a | 0.90%±0.40% a | 0.07%±0.09% b |
| norank_Gemmatimonadaceae | 1.34%±0.36% a | 0.95%±0.36% a | 0.21%±0.10% b |
| *Rickettsia* | 1.17%±1.26% a | 0.96%±0.99% a | 0.11%±0.13% a |
| other | 73.60%±28.82% a | 60.31%±31.81% a | 17.59%±13.56% b |

**Table S6.** Network parameters and the potential keystone at different sites. Average degree is the number of edges on each node, representing how many other nodes (OTUs) in the network are connected with the given node. Path length represents the nearest distance between two nodes. Diameter is the largest distance between two nodes in a network. Clustering coefficient shows the extent a node is connected to its neighbors. (After exclude top 3% OTU) B: hemolymph (B1–B12); H: hepatopancreas (H1–H12); I: intestine (I1–I12).

| Network | Nodes/edges | Avg.Degree | Char.path length | Diameter | Avg.Clust.Coeff | Keystone species |
| --- | --- | --- | --- | --- | --- | --- |
| B | 1006/36325 | 36.108 | 4.462 | 10 | 0.819 | Acidobacteriota*_Bryobacter_*OTU1764*、*Actinobacteriota*_Bifidobacterium_*OTU2119 |
| H | 768/21161 | 27.553 | 4.125 | 12 | 0.653 | Firmicutes_NK4A214_group_OTU6194、Proteobacteria_*Steroidobacter*_OTU1706 |
| I | 172/913 | 5.308 | 4.408 | 11 | 0.707 | *Proteobacteria_Ralstonia_OTU11036、Proteobacteria_Serratia_OTU10991* |

**Table S8.** Network parametersat at three site. ‘Original’ represents network constructed from OTUs (shown in Fig 4B in the main text). ‘Exclude top 3% OTUs’ represents network constructed after excluding top 3% OTUs in each sites in Table S7.

| Sites | Network | Nodes/edges | Avg.dgree | Avg. triangles | Avg. Eccentrivity | Avg. ClusteringCoefficiet |
| --- | --- | --- | --- | --- | --- | --- |
| Haemolymph | Original | 1006/36325 | 36.108 | 3277.71 | 8.52 | 0.82 |
|  | Exclude top 30 OTUs | 975/32665 | 33.503 | 2777.72 | 8.11 | 0.81 |
| Hepatopancreas | Original | 768/21161 | 27.553 | 2956.54 | 8.16 | 0.65 |
|  | Exclude top 23 OTUs | 224/1681 | 22.558 | 1880.47 | 8.71 | 0.64 |
| Intestine | Original | 172/913 | 5.308 | 48.21 | 7.35 | 0.71 |
|  | Exclude top 5 OTUs | 72/160 | 4.552 | 34.09 | 7.19 | 0.70 |


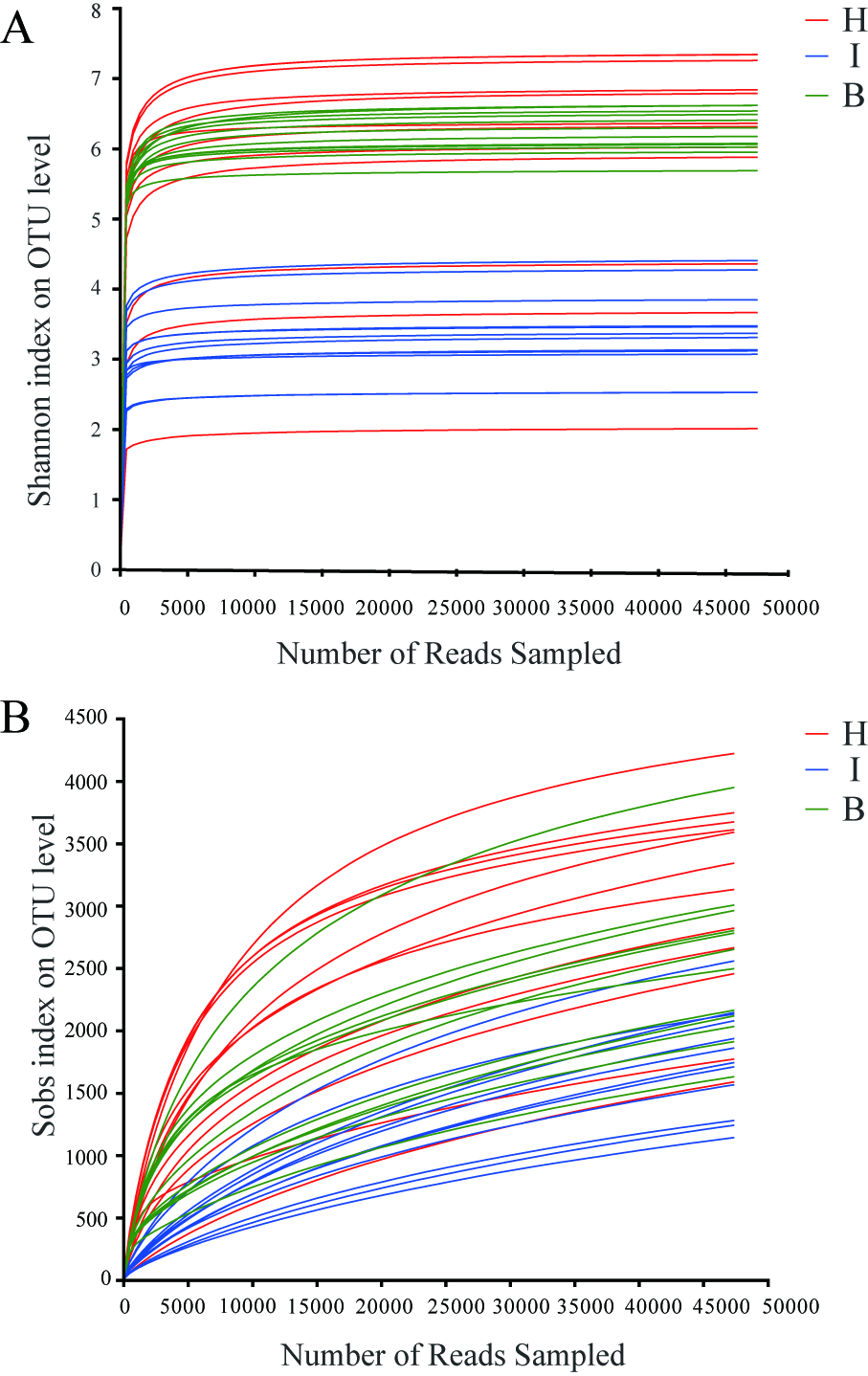


**Figure S1.** Rarefaction analyses of OTUs clustered at 97% sequence identity of all samples: **(A)** Shannon curves on OTU level at different sites. **(B)** Sobs curves on OTU level at different sites. B: hemolymph (B1–B12); H: hepatopancreas (H1–H12); I: intestine (I1–I12).


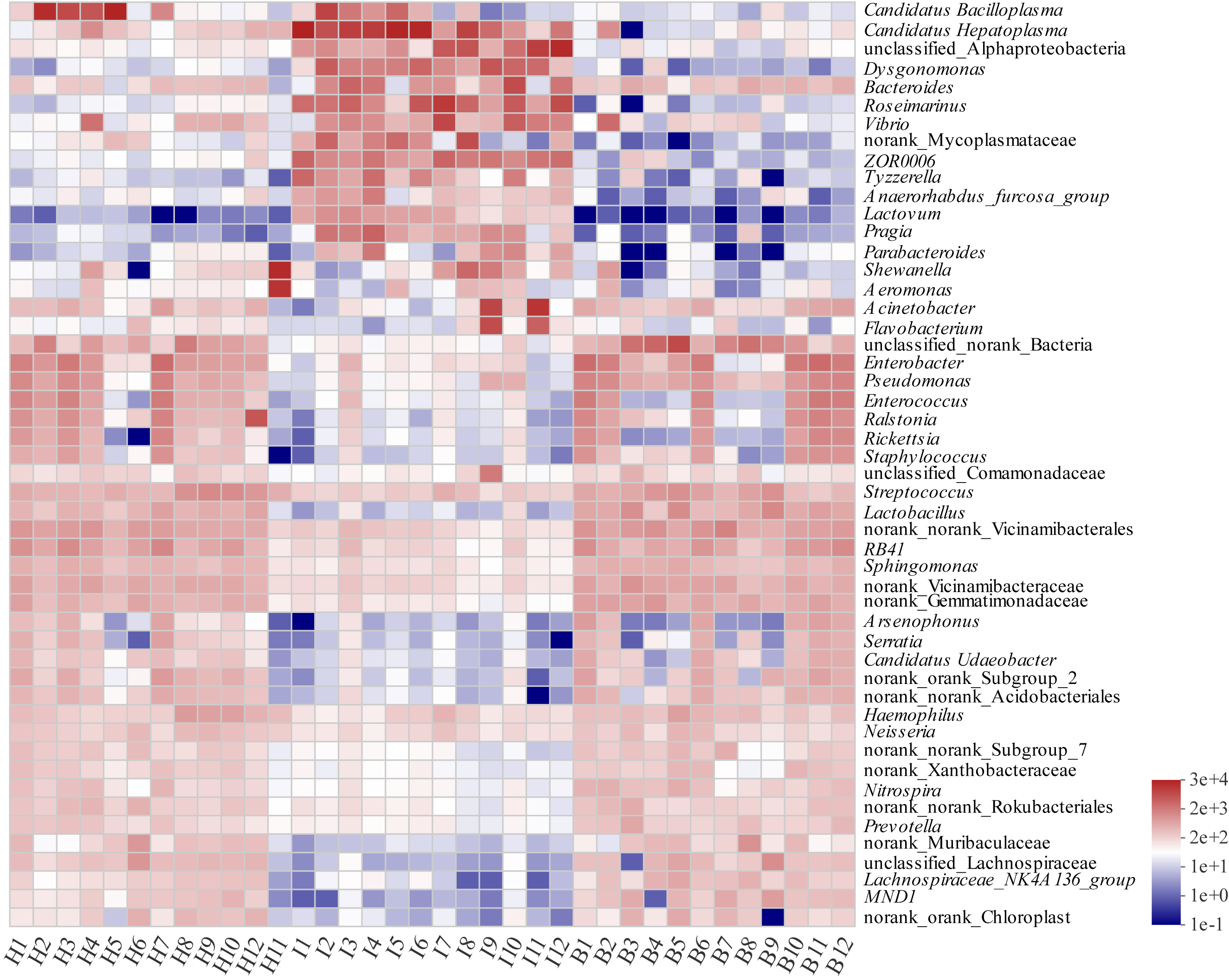


**Figure S2.** Heatmap showing the relative abundance of the bacterial communities at different sites (at genus level). The colored blocks indicate the percentage of sequences in each sample. B: hemolymph (B1–B12); H: hepatopancreas (H1–H12); I: intestine (I1–I12).


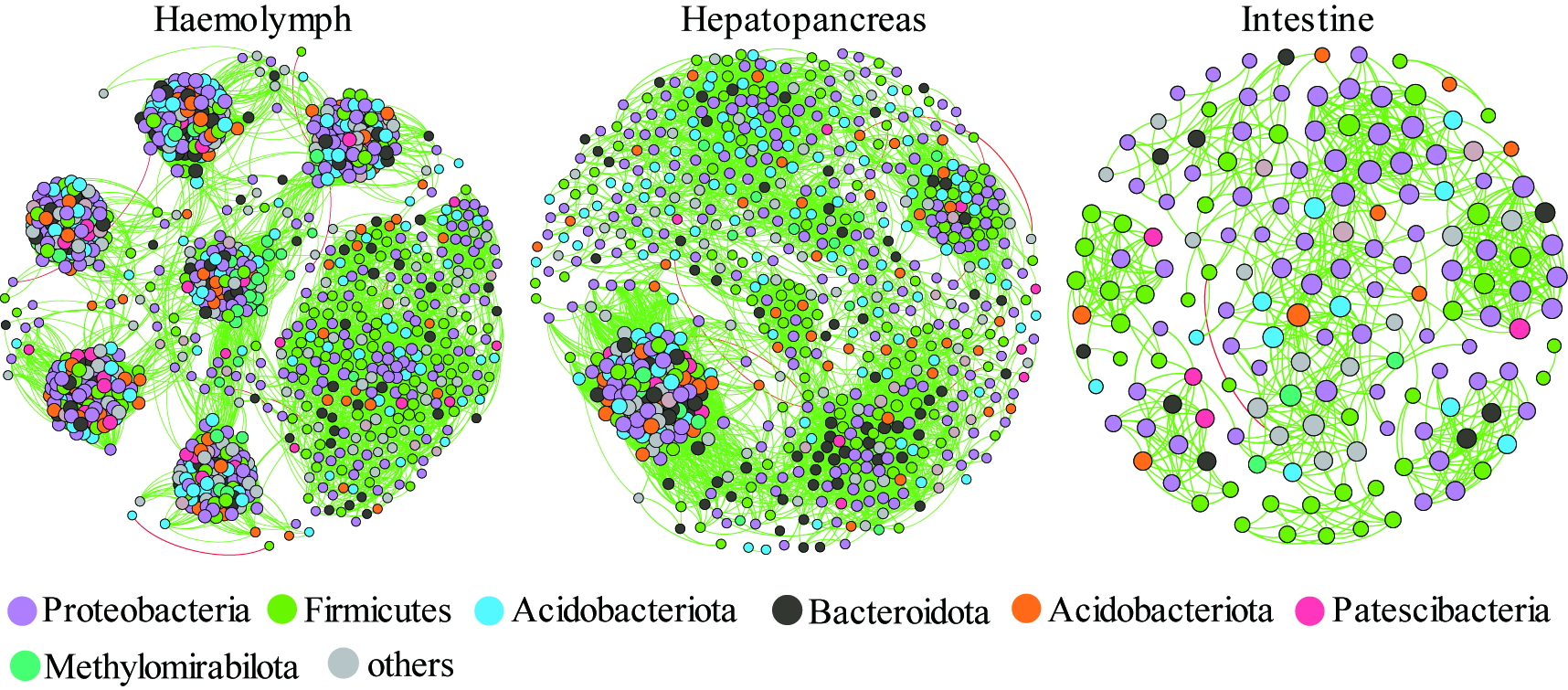


**Figure S3.** Network of co-occurring bacterial OTUs across three sites. Only Spearman’s correlation coefficient (r >0.7 or r < − 0.7 significant at *p* < 0.05) is shown. The nodes are colored according to phyla. Green edges represent positive correlations and red edges represent negative correlations. Node size is proportional to the betweenness centrality of each OTU, and edge thickness is proportional to the weight of each correlation. (After exclude top 3% OTU)


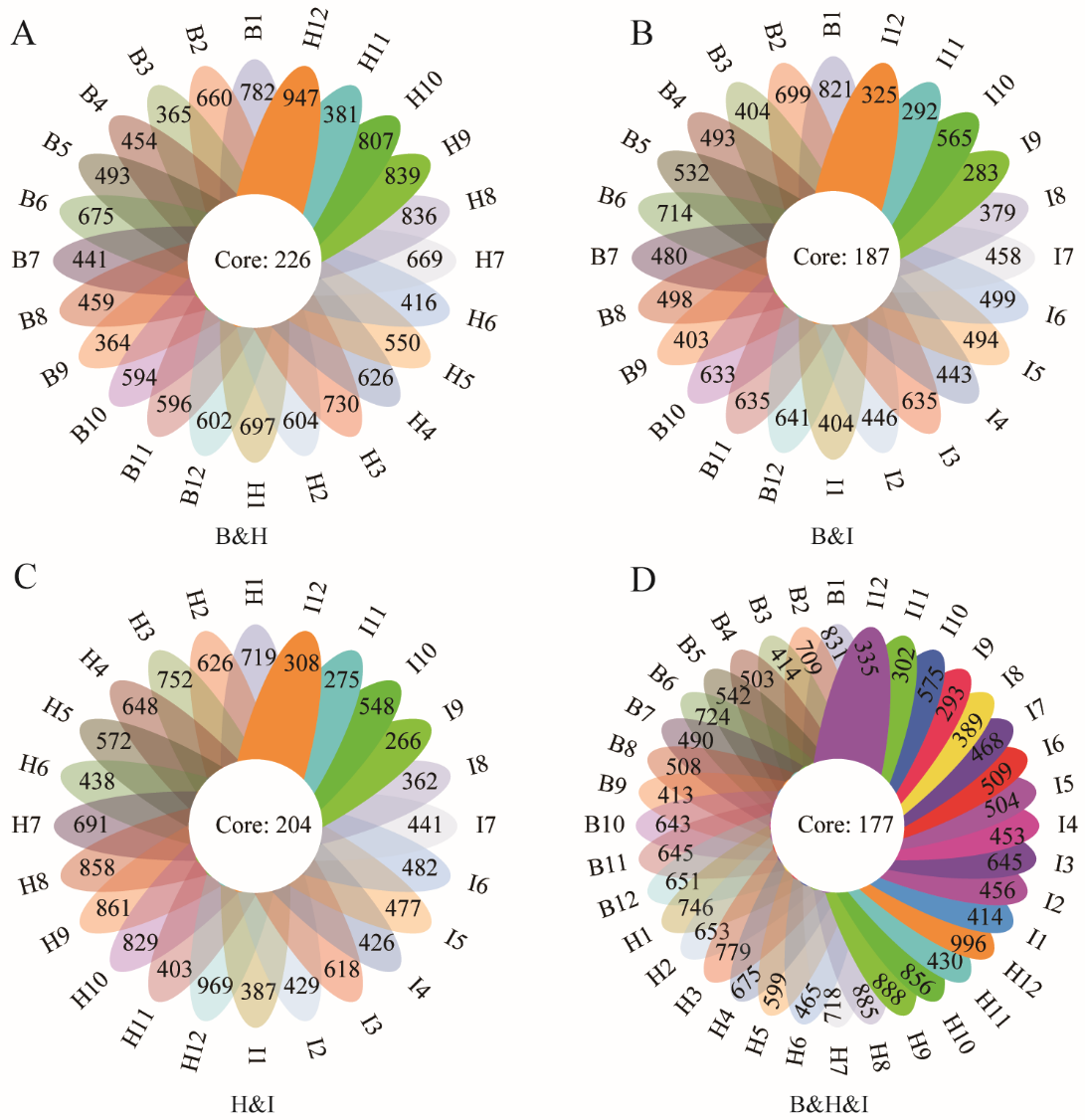


**Figure S4.** The petalogram shows the numbers of genera that were shared at different sites; **(A)** Number of genera that were shared between hemolymph and hepatopancreas. **(B)** Number of genera that were shared between hemolymph and intestine. **(C)** Number of genera that were shared between hepatopancreas and intestine. **(D)** Number of genera that were shared among hemolymph, hepatopancreas and intestine. B: hemolymph (B1–B12); H: hepatopancreas (H1–H12); I: intestine (I1–I12).
